# A Visual Metaphor for Oral Health Education in Hemodialysis Patients: A Pilot Study

**DOI:** 10.1111/scd.70088

**Published:** 2025-08-21

**Authors:** Gerhard Schmalz, Jonas Friedrich, Albert Stoeffler, Daniel Patschan, Stefan Büchi, Thomas Ebert, Deborah Kreher, Jonathan de Fallois

**Affiliations:** ^1^ Department of Conservative Dentistry and Periodontology Brandenburg Medical School (MHB) Theodor Fontane Brandenburg Germany; ^2^ Department of General Medicine University of Leipzig Leipzig Germany; ^3^ Department of Nephrology Rheumatology and Endocrinology University of Leipzig Leipzig Germany; ^4^ Department of Cardiology Angiology and Nephrology Klinikum Brandenburg Medizinische Hochschule Brandenburg Brandenburg Germany; ^5^ mediX Gruppenpraxis Rotbuchstrasse Zürich Switzerland

**Keywords:** hemodialysis, kidney replacement therapy, oral health, oral health education, oral health‐related quality of life

## Abstract

**Aims:**

The aim of this study was the comparison of PRISM (Pictorial Representation of Illness and Self‐Measure) with an interview‐based oral health education during a 4‐week follow‐up.

**Methods and Results:**

Patients under chronic hemodialysis (HD) were recruited and randomly assigned either to PRISM or a control group. At baseline, after the first intervention (T1), after 2 weeks (second intervention, T2) and after 4 weeks (third intervention, T3), three parameters were evaluated: gingival inflammation by papilla bleeding index (PBI), patient perspective (specific questionnaire), Oral health impact profile (OHIP‐G5). Fifteen patients each group finished the follow‐up. In PRISM group, neither after two (T2, *p* = 0.23) nor after 4 weeks (T3, *p* = 0.63), significant differences in PBI were found. In the Control, PBI values significantly increased between baseline and T3 (0.71 ± 0.52 vs. 0.95 ± 0.54, *p* = 0.02). Mainly non‐significant changes in the specific questionnaire were observed. The sum score of OHIP‐G5 did not differ between time points. Regarding the question whether the taste has worsened, a decrease was found in the control between T0 and T2 (*p* = 0.04). In the PRISM group, this decrease was apparent between T0 and T2 (*p* = 0.04) as well as between T0 and T3 (*p* = 0.04).

**Conclusion:**

Oral health education in HD patients appears challenging and offers a need for novel patient‐centered approaches.

**Abbreviations**
HD: hemodialysis
OHIP: oral health impact profile
OHRQoL: oral health‐related quality of life

## 1 Introduction

Patients under chronic hemodialysis (HD) have repeatedly been reported to suffer from a high prevalence of oral diseases [1]. This is caused by a risk complex, which is related to the underlying disease; chronic kidney diseases lead to decreased salivary flow, increased pH and phosphorus concentration in saliva [2]. Although not completely clarified, immunological changes appear to connect kidney diseases with oral inflammation [3]. Moreover, low level of education, diet, medication and insufficient oral care are risk factors for poor oral health in patients under HD [4]. Overall, those parameters can explain the high prevalence of caries and periodontal diseases, as already reported previously [3, 5].

One important point is often not considered: the behavior and the individual patient perspective. Previous research on oral health‐related quality of life (OHRQoL) of patients under HD indicate some conspicuous issues in this context [6]. Thereby, oral health complaints affect the OHRQoL of those patients. Moreover, the oral health perception is influenced by the burden of general disease and its psychosocial impacts [6]. Thereby, patients often do not perceive their poor oral health as a problem and/or feel unable to change the situation; this appears similar as a response shift in oral health perception, as presumed previously [6, 7]. Accordingly, missing education on oral health, the relevance of oral health behavior as well as low sensibilization for oral health issues appear a relevant factor, which requires appropriate patient‐centered concepts to improve their situation. Data on oral health education approaches for HD patients are still missing.

One previous study on patients prior to joint replacement surgery used a novel approach following a patient‐centered approach [8]. This approach, called Pictorial Representation of Illness and Self‐Measure (PRISM), is a visual metaphor, which originally was developed in psychosomatics medicine [9]. This visual metaphor is rather a self‐reflection task than a “classic” education. However, in the patient group before endoprosthesis, PRISM reached the same level of oral health education as a flyer‐based verbal briefing and led to positive effects beyond dentistry [8]. Due to the fact that the visual metaphor is a completely novel strategy for oral health education, it is still not investigated in patients under HD.

Taken together, this pilot study aimed in the comparison of PRISM with a “classic” oral health education, based on a standardized interview. Thereby, patients should be evaluated during a 4‐week follow‐up regarding clinical signs of gingival inflammation as well as the patient perspective (questionnaire and OHRQoL). Three hypotheses were formulated: (I) PRISM and the verbal interview lead to a reduction in oral inflammation (papilla bleeding index) during the study period. (II) PRISM leads to a higher effect on the self‐perceived relevance of oral health‐related topics than the verbal interview. (III) PRISM and the verbal interview lead to a similar change in OHRQoL.

## 2 Methods

### 2.1 Study Design

This current study was a prospective observational study with two different groups. The study design was reviewed and approved by the local ethics committee (306/22‐ek). A written and verbal information about the study, with a subsequently provided informed consent was performed for each participant. The whole study protocol was in line with the Declaration of Helsinki.

### 2.2 Sample Size

This current examination was a first pilot study to test PRISM as a novel instrument of patient education in HD. Focusing on the PBI as main outcome, for an effect size δ = 1 with a power of 80% and a significance level of <0.05, a sample size of at least 13 individuals per group was needed. To compensate potential drop‐outs, 15 patients per group should be included. The drop‐out rate was expected to be very low, because the patients regularly attended the dialysis center and the interviews and oral health examinations were performed within those regular visits. Another reason why the drop‐out rate was expected to be low was the short observational time.

### 2.3 Patients and Groups

Between April 1, 2023 and October 31, 2023, patients were recruited during their regular HD appointment in the Department of Nephrology, University of Leipzig, Germany. Mandatory criteria for inclusion in the current study were age of at least 18 years, chronic HD therapy and ability to provide informed consent. The following exclusion criteria were formulated:

- worse overall health status, making an oral examination impossible
- infectious diseases
- cancer
- dementia
- insufficient German language skills, which would not allow an appropriate interview and/or answering of the questionnaires

After information about the study and checking the in‐ and exclusion criteria based on the medical history of the patients, participants gave their informed consent for participation. Afterward, they were randomly assigned to one out of two groups. Within the randomization process, a box with lots was prepared, out of which the respective lot was drawn for each patient, allocating the participant either in the PRISM or control group. This was performed by an independent person, who did neither perform the interviews nor oral examinations. After eight participants were allocated to each of the two groups, age and gender were considered in the further randomization process to ensure comparable groups.

### 2.4 PRISM Group

**FIGURE 1:**
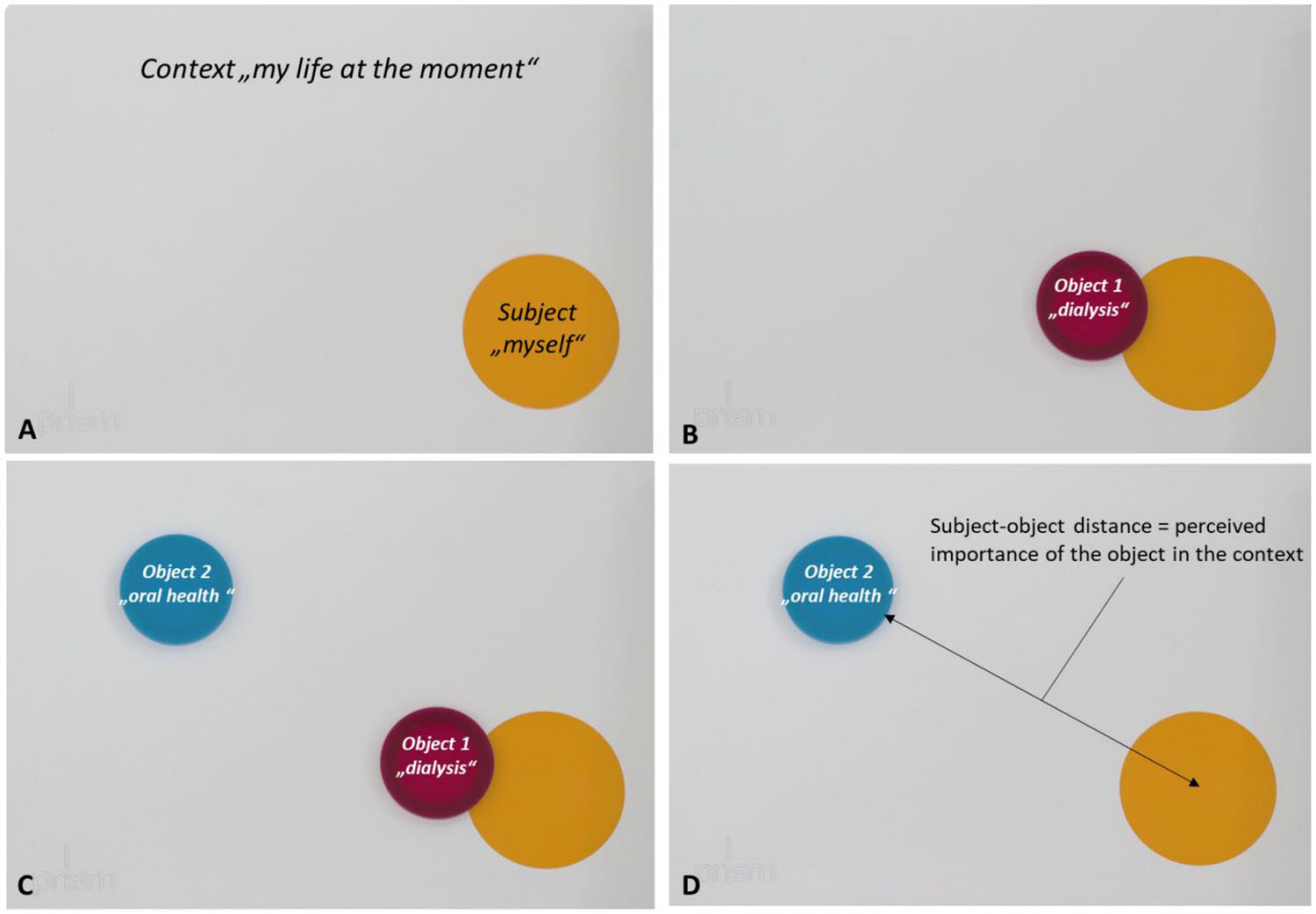
Example for the principle of PRISM for oral health education in the current study. (A) PRISM was explained to the patient, the metal board represents the context and the yellow circle the subject. (B) The patient was instructed to place an object reflecting his/her dialysis therapy in relationship to him/herself. (C) The patient was instructed to place a second object, reflecting the oral health. (D) The dialysis object was removed from the board to visualize the distance and thus lowly perceived importance of oral health in the view of the patient.

This group was educated using a visual metaphor, which is called PRISM, originating from the field of psychosomatics [9]. This method has been previously introduced in education of patients prior to endoprostheses in a similar way [8]. Within this visual metaphor, a white metal board (210 × 297 mm) is used as a predefined context, that is “my life at the moment” in the current study. In the bottom right‐hand corner of the board, a yellow circle (7 cm in diameter), which stands for the Subject (“myself”), is fixed. In relationship to this Subject, the patient can place differently colored object discs (5 cm in diameter). In this study, PRISM was used to visualize the subjectively perceived role of oral health for the individual patient. An example is given in Figure 1. The PRISM task was performed based on a clear interview manual, in which the interviewer has been experienced and calibrated previously. The interview lasted about 10 min.

### 2.5 Control Group

As a control group, an oral health education was performed based on a written and structured interview manual. This education was standardized and performed by the same calibrated interviewer as the PRISM tasks. The education included information on the relationship between oral health and kidney diseases, as well as information and instructions on appropriate oral hygiene and oral health behavior. The education lasted about 10 min.

### 2.6 Oral Examination

All participants underwent an oral examination at the beginning of the study by two experienced and trained dentists. Thereby, the number of remaining teeth, periodontal treatment need (based on periodontal screening index) and dental treatment need (defined as the presence of at least one carious lesion with cavitation) were evaluated. Moreover, it was checked whether patients wore their dental prostheses.

To evaluate the degree of gingival inflammation, the papilla bleeding index (PBI) was assessed. With a periodontal probe (PCP 15, hu friedy) the gingiva was touched and as the result, a graduated bleeding, ranging between score 0 (no bleeding/inflammation‐free gingiva) and 4 (profuse bleeding/severe inflammation) was recorded [10].

### 2.7 Questionnaires

#### 2.7.1 Questionnaire Regarding Oral Health and Kidney Disease

Based on a previous questionnaire, which was composed for patients prior to endoprosthesis [8], a modified form for patients under HD was composed. Thereby, different issues related to the patients relationship with their HD therapy, oral health condition and whether patients aimed in an improvement of their oral health were assessed. The questionnaire was answered between 0 (don't agree) and 5 (fully agree). This questionnaire has not been completely validated prior to the study, but a pre‐test was performed. During this, in the first step, systemically healthy individuals (*n* = 5) answered the questions and afterward, they discussed the understandability of the questions with the examiners. Similarly, HD‐patients (*n* = 5) answered the questionnaire and provided their feedback. On that basis, the final questionnaire was elaborated.

#### 2.7.2 OHRQoL

The German short form of the oral health impact profile (OHIP‐G5) [11] was used to evaluate the oral health‐related quality of life (OHRQoL). Within the OHIP‐G5, five questions regarding perceived impacts related with teeth, mouth, or dentures were rated with a score as follows: very often = “4”, fairly often = “3”, occasionally = “2”, hardly ever = “1”, and never = “0”. The lower the score, the better the perceived OHRQoL of the patient.

#### 2.7.3 Study Flow‐Chart

**FIGURE 2:**
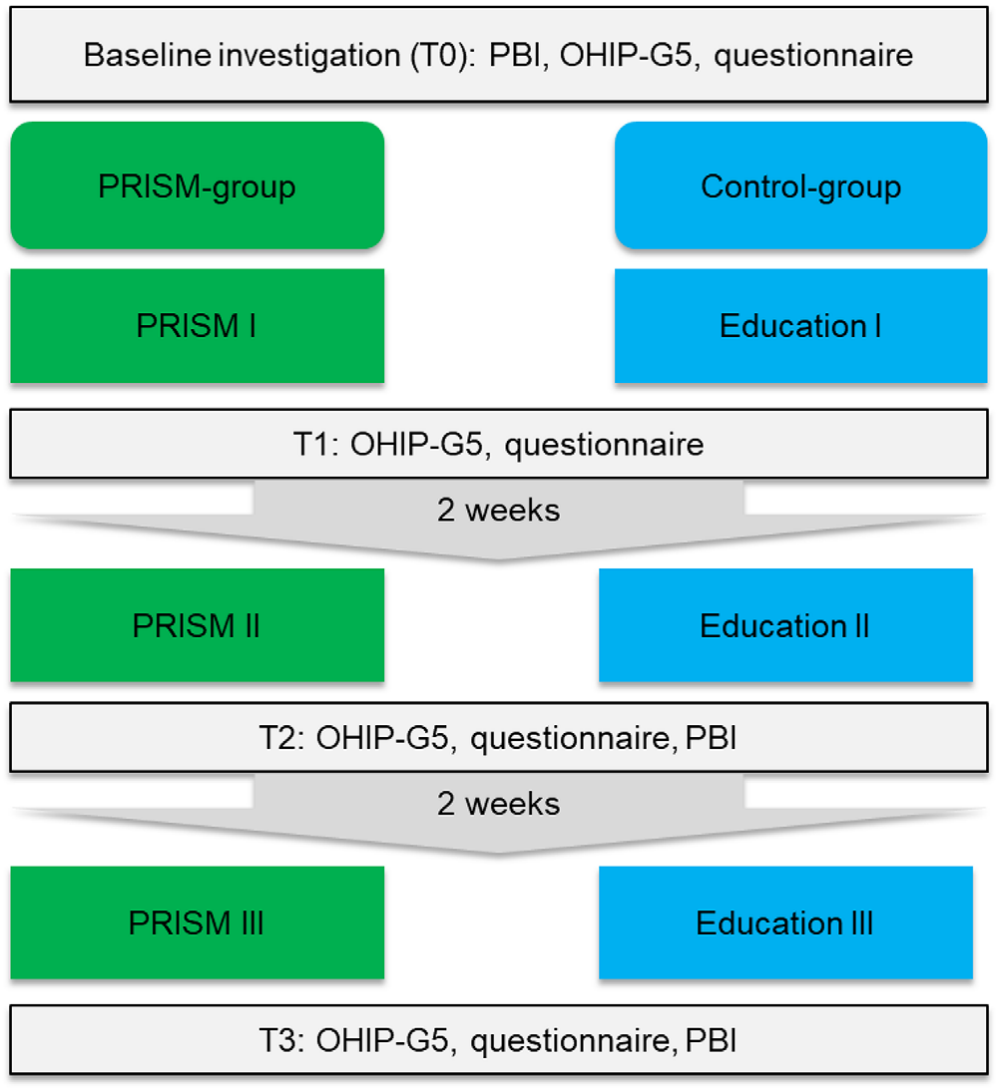
Study flow of this current study.

After information and informed consent, patients underwent the oral examination and answered the questionnaires for the first time (baseline). After this, patients were randomly allocated to their group and received their respective first interview (either PRISM or manual based). Immediately after the first interview (T1), the questionnaires were answered again. After 2 weeks, the second interview was performed, followed by the evaluation of PBI and questionnaires (T2). Finally, after another 2 weeks (4 weeks after baseline), interviews, PBI and questionnaires were assessed again (T3). After this, the observational period ended. The study flow is summarized in Figure 2.

### 2.8 Statistical Analysis

All analyses were conducted by using SPSS for Windows, version 24.0 (SPSS Inc., US). Firstly, Shapiro–Wilk‐test was performed, showing that PBI, age, and number of remaining teeth were normally distributed, while the other parameters were non‐normally distributed. Comparing two independent, normally‐distributed samples, *t*‐test was applied. Non‐normally distributed samples were compared by Mann–Whitney‐*U* test. More than two independent samples were tested by Kruskal–Wallis test. Categorical or nominal data were compared by Chi‐square test, respectively. The significance level was set at *p* < 0.05.

## 3 Results

### 3.1 Participants

**FIGURE 3:**
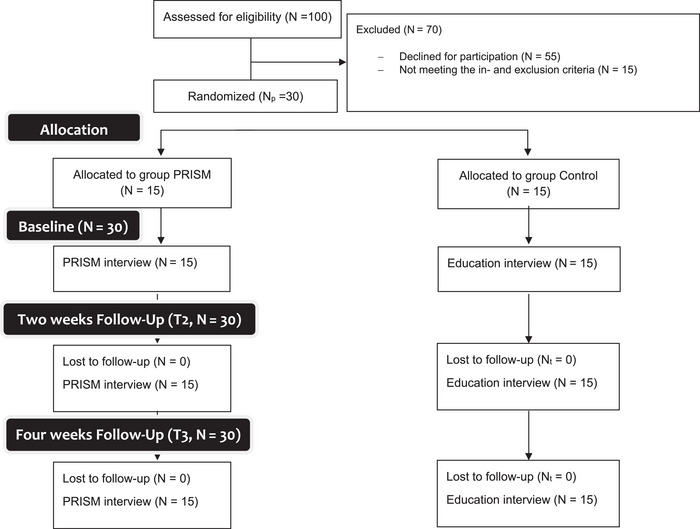
Participant flow through the study according to Butcher and Monsour [12].

Fifteen patients each group finished the follow‐up, without drop‐outs during the study period (Figure 3). Between groups, age, gender, and smoking habits were comparable. Similarly, there were no significant differences between groups regarding remaining teeth and treatment need (Table 1). The PBI at baseline was comparable between both groups (*p* = 0.28).

**TABLE 1:** Patient characteristics and oral health conditions of the two groups.

parameter	Control (*n *= 15)	PRISM (*n* = 15)	*p* value
**Gender (male) [*n* (%)]**	8 (53)	8 (53)	0.99
**Age in years (mv ± sd)**	60.73 ± 16.64	60.87 ± 19.21	0.98
**Smoking habits [*n* (%)]**			
Smoker > 10 cig./day	3 (20)	4 (26)	0.86
Smoker ≤ 10 cig./day	1 (7)	1 (7)	
non‐smoker	11 (73)	10 (67)	
**Oral conditions [*n* (%)] or (mv** ± **sd)**			
Number of remaining teeth	21.27 ± 8.48	21.67 ± 6.47	0.88
Periodontal treatment need	12 (80)	14 (93)	0.60
Dental treatment need	9 (60)	10 (67)	0.99
Dental prosthesis	4 (26)	3 (20)	0.99
Time under hemodialysis in years (mv ± sd)	5.4 ± 4.8	5.3 ± 4.7	0.78

### 3.2 PBI Values During Study Period

**FIGURE 4:**
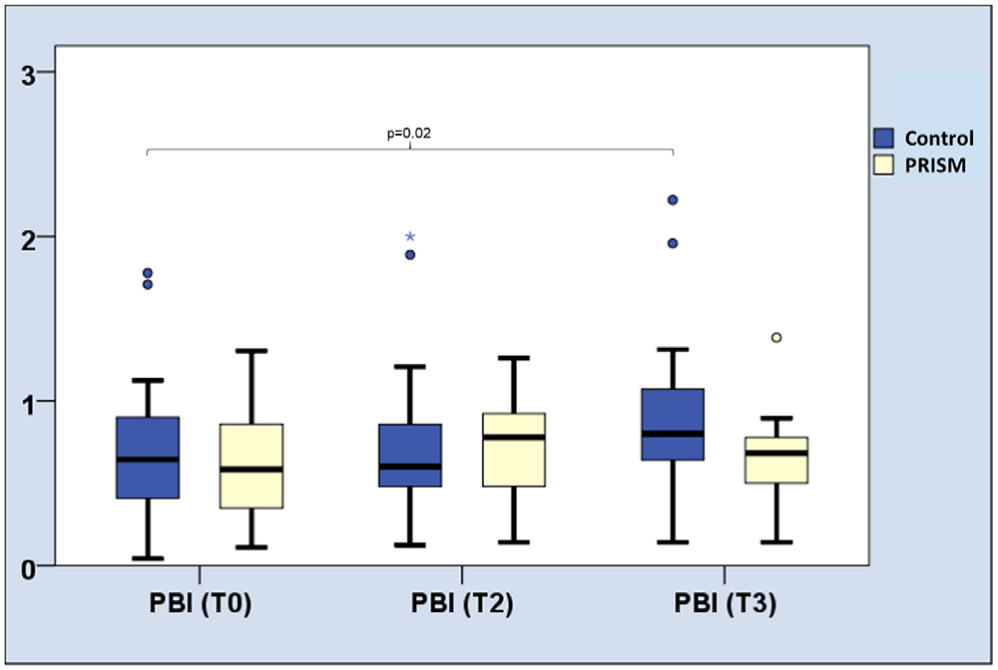
PBI values during the study period between the two groups.

In PRISM group, neither after two (T2, *p* = 0.23) nor after 4 weeks (T3, *p* = 0.63), significant change in PBI were found (Table 2). Between baseline and T3, PBI values slightly but significantly increased in the control group (0.71 ± 0.52 vs. 0.95 ± 0.54, *p* = 0.02). This was not apparent between baseline and T2 (Table 2, Figure 4).

**TABLE 2:** PBI values during the study period in the two groups, given as means and standard deviation.

Time point PBI	Control (*n *= 15)	PRISM (*n* = 15)
Baseline	0.71 ± 0.52	0.61 ± 0.37
T2 (after 2 weeks)	0.75 ± 0.56	0.72 ± 0.33
T3 (after 4 weeks)	0.95 ± 0.54	0.65 ± 0.30
*p* value baseline versus T2	0.77	0.23
*p* value baseline versus T3	**0.02**	0.63

### 3.3 Questionnaire Regarding Oral Health and Kidney Disease

Table 3 shows the changes in perception of patients regarding oral health‐related issues and their perceived relevance in context of HD or kidney disease, respectively. The median values of the questionnaires can be seen in Table S1. In both groups, a significant change in perception of the question “HD therapy is a part of me” occurred between T0 and T3 (*p* < 0.01). Only in the control group, the perception of the question “I see a relationship between my oral health and kidney disease” changed between T0 and T3, whereby a significantly higher perception was revealed (*p* = 0.01).

**TABLE 3:** Results for the differences in questionnaire results between time points.

Question	Control (*n* = 15)			PRISM (*n* = 15)		
	∆T0–T1	∆T0–T2	∆T0–T3	∆T0–T1	∆T0–T2	∆T0–T3
HD therapy is a part of me	0.27 ± 0.88	0.33 ± 0.82	0.67 ± 1.23^a^	0.13 ± 1.13	0.40 ± 0.83	0.73 ± 0.96^a^
My oral health is important in my life	0.13 ± 0.35	0.20 ± 0.56	0 ± 0.85	0.13 ± 0.64	0.33 ± 0.72	0.40 ± 0.91
My teeth are a part of me	0.13 ± 0.52	0.13 ± 0.83	0.07 ± 1.10	−0.27 ± 0.70	−0.07 ± 0.70	−0.27 ± 1.49
My teeth are a disturbing factor	0 ± 0.38	−0.20 ± 1.21	0 ± 0.93	−0.13 ± 1.96	−0.20 ± 1.15	−0.40 ± 1.45
I see a relationship between my oral health and kidney disease	0.67 ± 1.35	0.87 ± 1.88	1.27 ± 2.02^a^	−0.27 ± 2.34	0.20 ± 1.70	−0.13 ± 1.92
Since I have a kidney disease, my oral health is less relevant in my life	−0.20 ± 1.21	−0.60 ± 2.67	−0.40 ± 1.72	0.20 ± 2.11	−0.40 ± 2.35	−0.87 ± 1.85
Since I have a kidney disease, I have less energy for my oral health	−0.40 ± 1.35	−0.67 ± 1.99	−0.13 ± 1.73	0.40 ± 1.45	−0.33 ± 0.72	−0.53 ± 1.13
I made the decision to improve my oral health situation	−0.20 ± 1.08	0.47 ± 1.25	−0.13 ± 1.88	−0.27 ± 1.44	0.27 ± 1.39	0.47 ± 0.99

*Note*: Results are given as mean value ± standard deviation. Significant differences are highlighted.

^a^ *p* < 0.05.

### 3.4 OHRQoL

**FIGURE 5:**
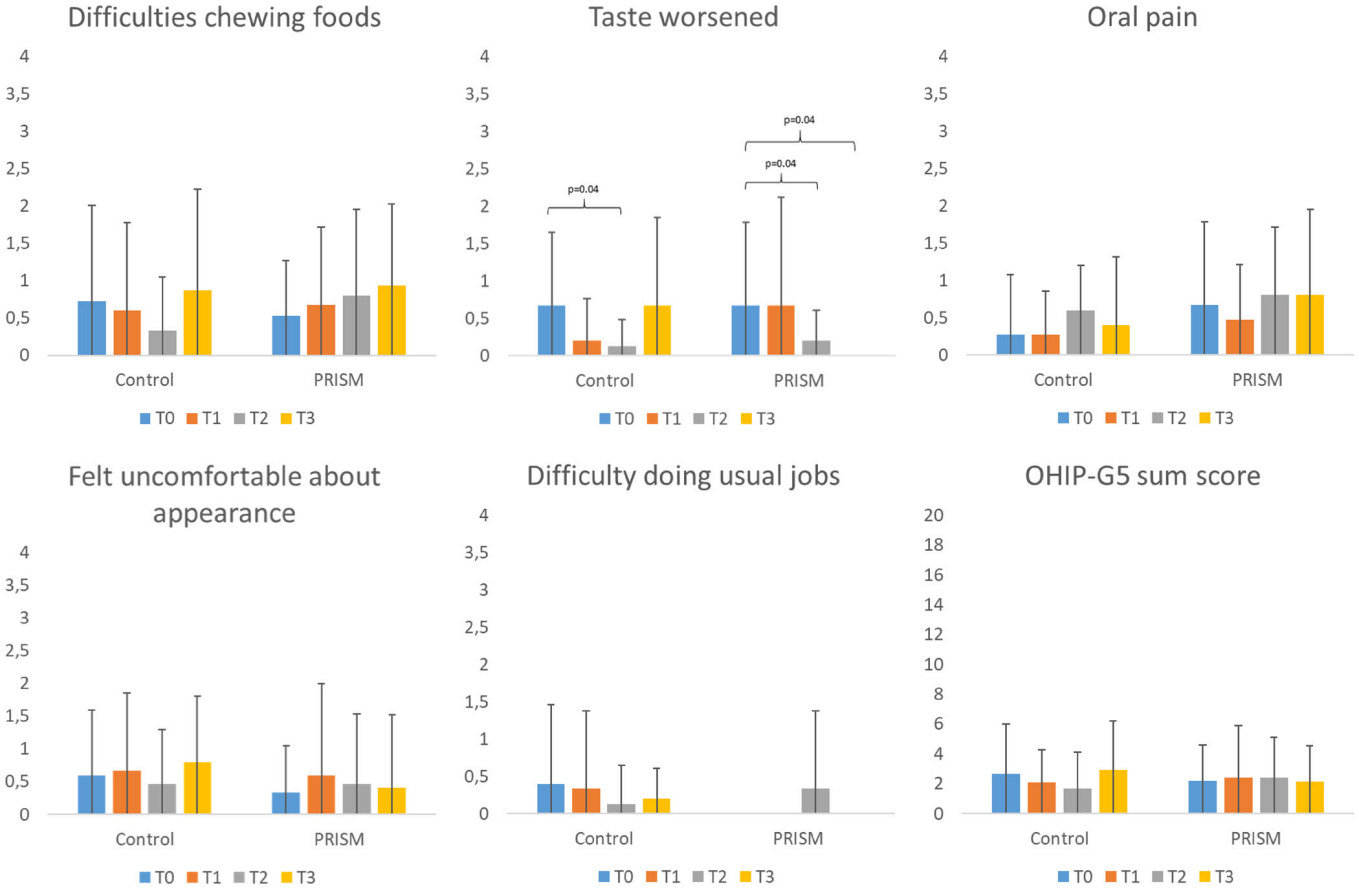
Results of the OHIP‐G5 given as mean and standard deviation.

The mean values of the OHIP‐G5 questions and the sum score are illustrated in Figure 5. Overall, the sum score of OHIP‐G5 did not differ between time points in both groups. Regarding the question whether the taste has worsened, a decrease (i.e., an improvement) was found in the Control between T0 and T2 (mean ± standard deviation: 0.67 ± 0.98 vs. 0.13 ± 0.35; median [IQR]: 0 [0–1] vs. 0 [0–0], *p* = 0.04). In the PRISM group, this decrease was apparent between T0 and T2 (0.67 ± 1.11 vs. 0.2 ± 0.41; 0 [0–1] vs. 0 [0–0], *p* = 0.04) as well as between T0 and T3 (0.67 ± 1.11 vs. 0; 0 [0–1] vs. 0 [0–0], *p* = 0.04).

## 4 Discussion

The main outcomes of this current pilot study show that neither PRISM nor the educational interview lead to a reduction of the clinical signs of gingival inflammation depicted with PBI. Moreover, no reasonable impact on the patient perspective was achieved over a 4‐week follow‐up.

Hypothesis I, that is, PRISM and the verbal interview lead to a reduction in oral inflammation (papilla bleeding index) during the study period was not confirmed. This hypothesis was formulated as it was expected that the intervention would lead to an improvement in oral hygiene behavior and thus in reduced gingival inflammation. With an average PBI mean of less than 1 (on a scale between 0 and 4), the degree of gingival inflammation appears quite low in the cohort and contradictory with the high periodontal treatment need. However, in a previous study, similarly low PBI scores were found in HD patients [13]. This previous study did also report deficits in oral hygiene, what is not in line with the low PBI values. Further studies evaluated the degree of gingival inflammation in HD patients with other indices, showing a high degree of gingivitis about 55% [14] or 62.14% [15]. This also appears somewhat contradictory and might indicate that the PBI would be not an ideal instrument in the HD cohort, as it might not appropriately reflect the status of gingival inflammation. Interestingly, the PBI increased slightly but significantly in the control group. This again reads suspicious, because during the intervention, an improvement was rather expected than a worsening. It is well described, that the PBI appropriately reflects gingival inflammation [16]; however, in this particular cohort, a slight increase in PBI could also be a result of more intensive oral hygiene. This remains speculative, but should be clarified in studies with a longer follow‐up.

Hypothesis II stated that PRISM would lead to a higher effect on the self‐perceived relevance of oral health‐related topics than the verbal interview. This hypothesis based on the experiences from a previous study in patients before endoprosthesis, which assessed only a pre‐post testing immediately after the intervention [8]. Obviously, PRISM did not have the expected effect, leading to the non‐confirmation of this second hypothesis. PRISM is a visual metaphor, which is personally salient for the patient and should lead to a self‐reflection [17]. Although it has primarily been developed for the visualization of suffering [18, 19], it has meanwhile been widely used for other purposes like suicidal intent [20], traveling‐related risk perception of tropical diseases [21], work‐related stress [22], or even undergraduate education [23]. In context of drinking, it has already been evaluated as an instrument to depict readiness to change behaviors [24]. This underlines the potential of PRISM as a tool within a patient‐centered approach. However, patients under HD are a distinct patient group. Patients with end‐stage kidney failure and particularly under HD suffer from a high disease burden, often causing hospitalization, depression, anxiety and low quality of life [25, 26, 27]. Thereby, the high disease burden might affect the potential to intervene with an oral health approach. This would explain the missing effect of both, PRISM and the verbal interview. On the other hand, the verbal interview led at least to an increased perception of the importance of oral health for patient´s kidney disease. This was not apparent for PRISM. Although PRISM is easy to understand and to perform [17], it might be somewhat difficult or confusing for the HD patient to understand the task and its context with oral health topics. Although the PRISM tasks were performed by an experienced and trained interviewer, which followed a clear structure, the patients potentially did not fully understand the task or particularly its link to oral health. Therefore, the combination of PRISM and the verbal interview might have a certain potential for future, as it might combine implicit with explicit information.

Finally, the hypothesis III, stating that PRISM and the verbal interview would lead to a similar change in OHRQoL was partly confirmed. Both interventions led to a slight change in one question, that is, whether the taste of foods had worsened. The sum score did not change, irrespectively of the intervention. Previously, a changed perception of OHRQoL by HD patients and thus a kind of response shift has been assumed [6]. The current study showed, that regardless of the remarkable dental and periodontal treatment need, a mainly unaffected OHRQoL was perceived by the patients. Therefore, it is unclear, what kind of effect can be expected due to the interventions. If one of the interventions would have been able to lead to an increased attention of the patients on their oral health situation, a worsening of OHRQoL during the study period might have been expectable. However, the OHRQoL remained widely unaffected by the respective intervention.

Strengths and limitations: overall, this pilot study addressed a clinically relevant and understudies issue. For the first time, oral health education was applied to HD patients in form of a verbal interview and using the visual metaphor PRISM. The study was performed by experienced interviewer and examiners. Nonetheless, several limitations require discussion. The sample size is quite low. However, HD patients are difficult to recruit and all patients fulfilled the follow‐up, what is remarkable in this severely ill cohort. Similarly, the short observational period is a limitation. Maybe, some effects would even occur after several months. Therefore, the current study was only a pilot study, whereby several points regarding sample size and follow‐up could be increased in a subsequent study. Similarly, the applied methodology must be recognized. The PBI appear to lead to a limited robustness in HD individuals, what was mentioned above. Additionally, the applied questionnaires might limit the findings, whereby the OHRQoL depicted by OHIP‐G5 seems also to bring limited information in a HD cohort. The specific questionnaire was composed based on a previous study and underwent a pre‐test, but the missing validation must be seen as a limitation, too. Moreover, the study was not blinded, as one of the clinical examiners was the interviewer. This must be seen as a bias of the study. The interviews and oral examinations during the HD in this vulnerable patient group were quite challenging and required experienced interviewers and examiners. Due to organizational reasons, it was not possible to ensure a strict separation between interviewer and examiner. This limits the study´s results. On the other hand, it was not possible to conceal which group‐dependent intervention was performed, because the patients see, whether they receive a visual metaphor or a checklist‐based interview. The PRISM task has been elaborated for the special patient group. Based on the limited effect in the current study, this might also be a point needing consideration. Potentially, a combination of PRISM and verbal interview would be an interesting test group. Due to the small number of patients, this was not performed in the current study, similarly as for a control group without any interventions. The monocentric design is a further limitation, making this pilot study not representative for patients under HD. Taken together, this pilot‐study addresses a very special field in context of patient‐centered dental interventions with a visual metaphor in a specific cohort of patients under HD. Thus, the results are not generalizable and must be interpreted against this background. However, testing this type of intervention in further studies might be of interest.

## 5 Conclusions

Neither PRISM nor a verbal oral health education led to improvement of gingival inflammation or relevant changes in oral health perception over 4 weeks. The combination of both approaches might be a future perspective, requiring further research. Overall, oral health education in HD patients appears challenging.

## Ethics Statement

This current study was designed as a monocentric cohort study, which has been reviewed and approved by the local ethics committee (306/22‐ek). All participants were informed about the study and gave their written informed consent. The authors confirm that all methods were performed in accordance with the relevant guidelines and regulations and were performed in line with the Declaration of Helsinki.

## Conflicts of Interest

The authors declare that they have no competing interests.

## Supporting information




**Supplementary Table 1**: Absolute values for the questionnaire at each time point. Values are shown as median (IRQ).

## Data Availability

The datasets used and/or analyzed during the current study are available from the corresponding author on reasonable request. The data are not publicly available, because of the Pseudonymization and data protection guidelines according to the ethics approval.
