# Supplementary material for: A Visual Metaphor for Oral Health Education in Hemodialysis Patients: A Pilot Study
Source: Spec Care Dentist. 2025 Aug 21;45(4):e70088. doi: 10.1111/scd.70088 (PMC12371229; doi:10.1111/scd.70088)
Supplement: Supplementary file 1 — Supplementary Table 1: Absolute values for the questionnaire at each time point. Values are shown as median (IRQ). [file SCD-45-0-s001.docx]

**Supplementary material**

**Supplementary table 1**: Absolute values for the questionnaire at each time point. Values are shown as median (IRQ).

| Question | **Control (n=15)** | | | | **PRISM** **(n=15)** | | | |
| --- | --- | --- | --- | --- | --- | --- | --- | --- |
|  | T0 | T1 | T2 | T3 | T0 | T1 | T2 | T3 |
| HD therapy is a part of me | 5 (3-5) | 5 (4-5) | 5 (4-5) | 5 (5-5) | 5 (3-5) | 5 (4-5) | 5 (5-5) | 5 (5-5) |
| My oral health is important in my life | 4 (3-4) | 4 (3-5) | 4 (3-5) | 4 (3-5) | 4 (3-5) | 4 (3-5) | 4 (3-5) | 5 (4-5) |
| My teeth are a part of me | 5 (4-5) | 5 (4-5) | 5 (4-5) | 5 (4-5) | 5 (5-5) | 5 (5-5) | 5 (4-5) | 5 (5-5) |
| My teeth are a disturbing factor | 0 (0-2) | 0 (0-2) | 0 (0-1) | 0 (0-1) | 0 (0-1) | 0 (0-1) | 0 (0-2) | 0 (0-2) |
| I see a relationship between my oral health and kidney disease | 0 (0-2) | 1 (0-4) | 3 (0-3) | 3 (0-3) | 0 (0-4) | 0 (0-3) | 1 (0-3) | 1 (0-3) |
| Since I have a kidney disease, my oral health is less relevant in my life | 1 (0-4) | 1 (0-3) | 0 (0-3) | 1 (0-3) | 1 (0-4) | 3 (0-4) | 0 (0-4) | 0 (0-2) |
| Since I have a kidney disease, I have less energy for my oral health | 1 (0-4) | 1 (0-3) | 1 (0-3) | 2 (0-4) | 2 (0-4) | 3 (0-5) | 1 (0-3) | 1 (0-3) |
| I made the decision to improve my oral health situation | 4 (3-5) | 3 (2-5) | 4 (4-5) | 4 (3-5) | 4 (3-5) | 4 (2-5) | 4 (3-5) | 5 (3-5) |
